# Compatibility of Whole Wheat-Based Composite Flour in the Development of Functional Foods

**DOI:** 10.17113/ftb.62.04.24.8588

**Published:** 2024-12

**Authors:** Amani Weerarathna, Matara Arahchige Jagath Wansapala

**Affiliations:** Department of Food Science and Technology, Faculty of Applied Sciences, University of Sri Jayewardenepura, Gangodawila, 10250 Nugegoda, Sri Lanka

**Keywords:** composite flour, whole wheat flour, sustainable alternative flour

## Abstract

Over the last decades, eating habits have shifted towards convenient foods with shorter preparation times due to people’s busy lifestyles and higher living standards. Rapid changes in dietary patterns and lifestyles with the industrialization and globalisation have led to the escalating incidence of chronic diseases, which has paved the way to greater interest in dietary changes regarding nutritional status and health benefits. Composite flour is a combination of wheat and non-wheat flours or exclusively non-wheat flour with improved nutritional value, therapeutic properties and functional characteristics. The application of composite flours in the food industry is an important milestone that maximises the use of indigenous crops while optimising the product quality, nutritional value, organoleptic properties and consumer acceptance. This paper provides a comprehensive overview of the suitability and compatibility of alternative composite flours in the food industry with regard to the existing formulations. Furthermore, the suitability of composite flours in food products in terms of nutritive and therapeutic value is emphasised. It was found that food products with higher nutritional and therapeutic value and acceptable sensory properties can be formulated by blending different non-wheat flour sources with wheat flour at different ratios. Composite flours have the potential to reduce the risk of non-communicable diseases, particularly type 2 diabetes, cardiovascular disease and cancer. It can be concluded that the use of composite flours in the food industry is a trending approach due to their numerous benefits.

## INTRODUCTION

Wheat, the staple food in many parts of the world, is responsible for around a fifth of the world’s calorie supply. This is virtually a quarter of the calorie intake from grains, which provide almost half of the calories consumed worldwide. The preference for wheat is based on its ability to serve as a basic ingredient in different products, particularly bread, noodles, pasta, cakes, pastries, crackers and flatbread products. Wheat is the most traded grain because its robustness and longer shelf life in the absence of humidity and rodents make it more suitable for transport and storage than any other commodity. The development of wheat consumption shows the displacement of staple foods that have been used as the main source of calories in different parts of the world. The key factors that describe the development of wheat consumption in developing countries are population growth, income growth, determination of the relative prices of wheat and other staple food and the preferences of end consumer. Drastic population growth and rising incomes significantly increase the demand and thus the wheat consumption. End consumer preferences are either determined or perhaps induced by technology, lifestyle and income. Furthermore, the increasing employment of women has encouraged the shift in dietary patterns towards convenient foods with less preparation time, thus favouring the use of wheat-based products (*1*).

Consumers prefer refined wheat-based products to whole grain wheat-based products because the texture, eating quality and taste of whole wheat-based products are less appealing (*2*). The removal of outer layers of whole wheat grains, which are rich in nutrients and bioactive compounds, during milling results in the loss of important health benefits of the grain. The refined wheat-based products have a high content of digestible carbohydrates with high glycaemic index (GI), low amounts of minerals, B-group vitamins, polyphenols, β-carotene and dietary fibre, and a low quantity and quality of proteins (*3*-*5*). The consumption of foods with high glycaemic index exacerbates the incidence of type 2 diabetes and other cardiometabolic diseases, which have been recognised as a major socio-economic burden worldwide. Therefore, immediate dietary interventions are needed to develop appropriate low GI foods with high palatability to effectively prevent and regulate type 2 diabetes and improve cardiometabolic health (*6*). Moreover, despite the increasing consumption of wheat-based products around the world, a wide spectrum of health complications associated with wheat intolerance have been reported (*7*). Three possible wheat-related disorders, namely wheat allergy (WA), coeliac disease (CD) and non-coeliac wheat sensitivity (NCWS), have been detected in susceptible individuals exposed to either wheat or wheat components, particularly wheat protein (*8*). Gluten, the primary storage protein in wheat, has been identified as the major culprit for the onset of wheat-related diseases (*7*). CD is a chronic autoimmune disease triggered by the ingestion of gluten and causes small intestinal mucosal damage in hereditarily predisposed individuals (*8*, *9*). The worldwide prevalence of CD based on a diagnosis confirmed by biopsy and serology is 0.7 and 1.4 % respectively (*10*). CD leads to villous atrophy, which flattens the villi in the small intestine and reduces the surface area for absorption of nutrients, leading to various complications such as malnutrition, micronutrient deficiencies and gastrointestinal symptoms, particularly bloating, nausea and abdominal discomfort (*7*). Although gluten is beneficial for maintaining the viscoelastic properties of baked goods, consumer interest has shifted from wheat-based products to wheat-substituted products due to the aforementioned consequences (*11*).

Recently, a rapidly growing demand for functional foods, also known as health-promoting and disease-preventing foods, has been observed as consumer awareness and interest in health and nutrition has increased (*12*). Fruits, vegetables, whole grains and legumes have been included in the diet because the link between diet and disease has been recognized to provide a substantial amount of bioactive components such as phytochemicals, dietary fibre and protein. These components impart specific physiological benefits to functional foods (*2*, *12*). There is growing evidence that regular ingestion of whole grains can reduce the risk of chronic degenerative diseases and obesity (*4*). The antioxidant capacity of whole grain phytochemicals, particularly polyphenols, is responsible for the alleviation of oxidative stress, resulting in the delayed onset of some chronic diseases (*13*). This has led to the consideration of fortification or substitution of whole wheat flour with other non-wheat cereal flours (*4*).

## COMPOSITE FLOUR TECHNOLOGY

Composite refers to the combination of two or more components with the aim of creating a novel product that is superior to the individual components in terms of improved properties, performance or economy (*14*). Since 1960, mixed flours or blends have been scientifically referred to as composite flours. Currently, composite flour is simply introduced as mixture or replacement of various types of flour with or without wheat flour (*15*).

The need for composite flour arose with the scarcity of wheat due to climatic or economic fluctuations and the development of scientific knowledge about the adverse health effects of wheat flour (*16*). Inadequate climate for wheat cultivation around the world has led to the extensive use of non-wheat grains in the bakery industry. For instance, rice in South and East Asia, maize in central and South America, millet and sorghum in Africa and rye and oat in Northern Europe. In addition to the aforementioned reasons, the occurrence of lower yields and inferior quality of wheat due to global climate change has encouraged the extensive use of other cereals that can be grown further than the borders of Africa and Asia due to global warming (*17*).

Moreover, the blending of wheat flour with non-wheat flour is economically and nutritionally beneficial (*16*). As far as economic importance is concerned, the main reasons for the greater interest in composite flours in developing countries are to save foreign exchange by reducing wheat imports and thus encourage local agriculture (*4*, *15*). Enhancing the value of domestic agriculture through better utilisation of local crops helps to reduce dependence on imported wheat and ultimately ensure food security (*4*). Composite flour products can be offered at affordable prices for low-income groups due to the use of cheaper substitutes (*18*). On the other hand, composite flours have a greater nutritional importance. Compared to wheat flour, they have a better nutritional profile with a high protein and vitamin content. Moreover, composite flours can be recognised as a healthier product for people suffering from malnutrition and health problems (*15*). In addition, the increasing prevalence of coeliac disease and other gluten-associated allergies has triggered the demand for non-wheat flour-based products (*4*). Each individual component of the composite flour has a characteristic colour, texture and nutritive value that can enhance its use in various food products (*15*). Although numerous studies have been carried out on the subject of composite flours, a greater number of studies have focused on partial substitution of wheat flour rather than the complete replacement.

## SUSTAINABLE ALTERNATIVES FOR COMPOSITE FLOURS

### Cereals

Maize and rice can be introduced as the most important cereal grains in the human diet alongside wheat (*4*). Rice is an important staple food for more than half of the world’s population, especially for Asians (*19*). Recently, researchers have focused more attention on different rice varieties due to their health benefits, high amounts of bioactive compounds and resistant starch (*20*). Rice is superior to other cereals due to its low sodium content, high content of easily digestible carbohydrates and hypoallergenic properties (*19*). The most important antioxidants in different rice varieties are polyphenols such as anthocyanins, proanthocyanidins and phenolic acids concentrated in red, black and white rice respectively (*20*). Pigmented rice varieties have anticancer (*21*), antidiabetic (*22*, *23*) and antioxidant (*21*) properties (*24*). Rice flour does not contain a unique wheat gluten protein and is often consumed by coeliac patients either as cooked rice or as flour. Black rice varieties have been identified by *in vitro* digestion as a suitable dietary intervention for coeliac patients due to their potential antioxidant and anti-inflammatory effects (*25*).

Maize is the third most important staple cereal consumed worldwide. It is considered a versatile crop that has many uses worldwide, including as livestock feed, for human consumption and other non-food purposes (*26*). Despite the fact that white and yellow maize varieties are mainly used for human consumption, an increasing consumption of pigmented varieties has been observed due to the functional properties, especially antioxidant properties of anthocyanins that are responsible for the colour of the coloured varieties (*27*). A study reported that combination of maize and whole wheat flour increased the ash, fat and fibre content of the composite flour without affecting the organoleptic properties up to 20 % of maize (*28*).

**Fig. 1:**
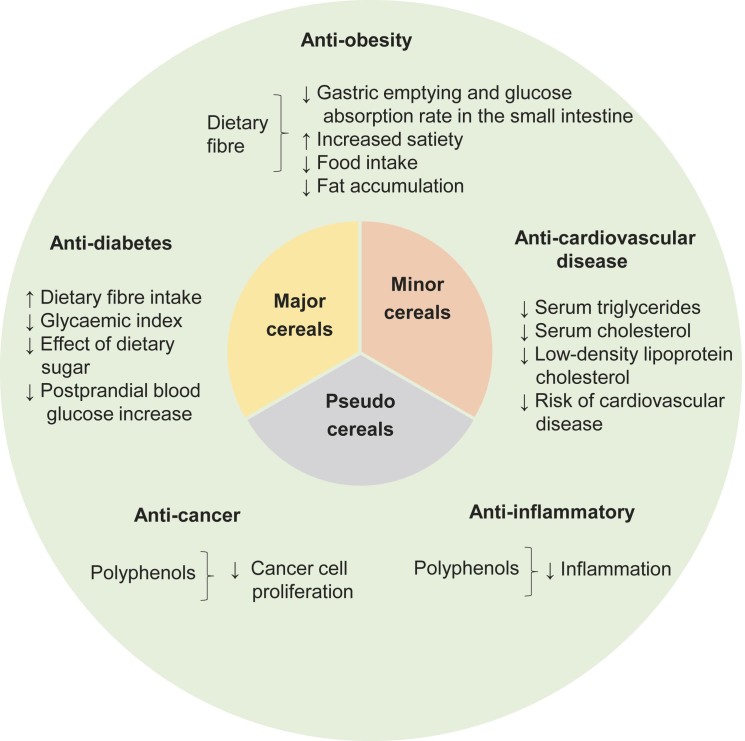
Health benefits of cereals and pseudocereals (*31*-*44*)

Although the major cereals contribute to more than 50 % of the global caloric demand, they are significantly deficient in phytonutrients and micronutrients, especially vitamins and minerals. On the contrary, certain minor cereals and pseudocereals, which are nutritionally equivalent or even superior to the major cereals, serve as excellent sources of phytonutrients and micronutrients (*29*). Minor cereals include sorghum, millet, barley and oats, while pseudocereals consist of amaranth, buckwheat and quinoa (*30*). The health benefits of cereals and pseudocereals are shown in Fig. 1 (*31*-*44*).

Millet and sorghum are known as important food crops in sub-Saharan Africa and south Asia (*30*). They are affordable, available and can have positive effects on human health and nutrition (*45*). Millets are considered as an important cereal with a high dietary fibre, micronutrient and phytochemical content. Finger, kodo, barnyard, little and pearl millet belong to the millet group (*46*). The addition of sorghum and pearl millet flour to the whole wheat flour in the production of certain flatbread products (*i.e.* chapattis and biscuits) leads to reduced GI of the products (*47*). Sorghum and millet enhance the hypoglycaemic effect of food products and play a crucial role in the regulation of hyperglycaemia (*45*-*48*). They are also beneficial in reducing serum triglyceride and cholesterol values (*49*, *50*), body mass management (*51*, *52*) and reducing the risk of gastrointestinal ailments (*30*, *53*, *54*). Barley is the fourth most important cereal worldwide (*55*). Barley and oats are ideal sources of β-glucan, water-soluble dietary fibre, with amounts of 2.5–16 and 2.3–8.5 %, respectively (*56*, *57*). β-glucans are able to lower glycaemic index and increase the insulin response in diabetic patients (*55*, *58*). They can also improve lipid metabolism, mitigate the occurrence of coronary heart disease by lowering plasma cholesterol (*55*, *59*) and reduce gastrointestinal disorders (*55*, *57*, *60*).

### Psuedocereals

Buckwheat is the well-known type of pseudocereals. It has higher protein (14.94 %), ash (1.855 %), total phenolic (expressed in gallic acid equivalents, 21.64 mg/g) and antioxidant content (expressed in Trolox equivalents, 131.36 mg/g) than whole wheat flour (11.92, 1.202, 5.66 mg/g and 15.91 mg/g, respectively) (*61*, *62*). The antioxidant activity of buckwheat is attributable to hyperin, rutin, quercetin and catechins, which have numerous health benefits (*63*). Buckwheat is able to reduce the risk of hypertension (*64*), hypercholesterolaemia (*65*) and diabetes (*65*) as it contains nutrients such as thiamin-binding proteins, flavonoids and proteins that can have favourable effect on the regulation of blood pressure, serum cholesterol and glucose level (*61*, *63*). Quinoa, also recognised as a ’complete food’, is an excellent source of minerals (calcium, iron, zinc and copper), vitamins (B_1_, B_2_, B_3_ and E), phytochemicals (phytosterols, saponins and phytoecdysteroids), unsaturated fatty acids and essential amino acids (methionine, lysine and threonine) (*66*). Due to its high fibre content, quinoa plays a crucial role in diets intended to reduce the risk of cardiovascular diseases and obesity. Quinoa flour also contains higher protein content (15.96 %) (*63*). Quinoa is known to have a favourable effect on human gastrointestinal (*67*), cardiovascular (*68*) and metabolic health (*66*, *69*-*71*). Amaranth flour has higher protein, vitamin (folate and B_12_) and mineral content (iron, magnesium, potassium and phosphorous) than whole wheat flour. Water-holding capacity (WHC), which depends on the interaction between fibre and protein, is higher in amaranth flour than in whole wheat flour. Viscosity, stability and textural properties of the products can be improved by high WHC of flours. Textural properties of amaranth show higher correlation with its pasting properties. The stability of the paste made from amaranth starch is higher than that of rice, corn, wheat and potato starch. Amaranth has the potential to increase antioxidant capacity, improve different immune parameters, mitigate blood pressure and reduce cholesterol when consumed frequently (*72*-*75*). Buckwheat and amaranth are categorised as low GI foods, while quinoa has a medium GI. Therefore, these pseudocereals can be used as functional ingredients in novel product formulations to regulate the glycaemic impact of the final product (*61*).

### Legumes

**Fig. 2:**
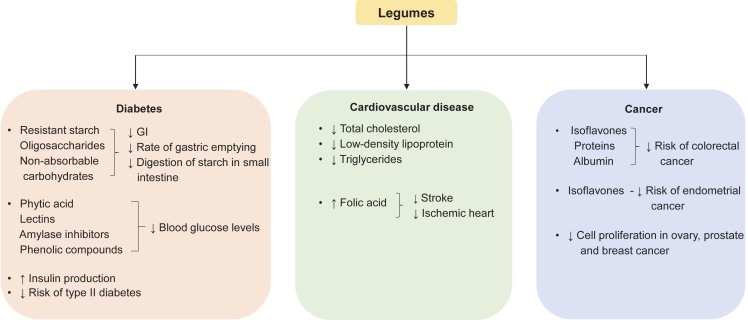
The therapeutic importance of legumes (*83*-*88*). GI=glycaemic index

In contrast to the past, the demand for functional foods with increased nutritional value, *e.g.* products that can fulfil the nitrogen and amino acid requirement of the body, has increased due to people’s health awareness. Meanwhile, the consumption of plant proteins instead of animal proteins has become a global trend in the context of environmental conservation and the reduction of gas emissions (*76*). Dry beans, chickpeas, dry peas, lentils, cowpeas and broad beans are important crops belonging to legume family (*77*). Flours made from pulses are used as supplements of wheat flour due to their higher protein content (*76*). Wheat flour is blended with pulses to improve the balance of essential amino acids and thus the protein quality, as cereal proteins are deficient in lysine but rich in sulphur-containing amino acids, especially methionine and cysteine, while legumes are a rich source of lysine but deficient in sulphur-containing amino acids (*78*). Yellow peas, green peas, red lentils and chick peas have a higher protein content (19.82, 21.78, 23.72 and 22.19 %) and a higher ash content (2.32, 2.38, 2.10 and 2.66 %) than whole wheat flour (15.13 and 1.80 %) (*79*). People suffering from hypertension (*80*) and hyperglycaemia (*81*) benefited from the consumption of bean flour due to the phenolic compounds that can inhibit enzyme activity (*82*). Moreover, chickpea flour has been recognised as a hypoglycaemic agent that plays a crucial role in the regulation of type 2 diabetes due to the presence of a proteinaceous fraction and specific polysaccharides identified as potent inhibitors of the enzyme α-amylase (*82*). The therapeutic importance of legumes is shown in Fig. 2 (*83*-*88*). Legumes are often overlooked because of the antinutritional factors. Nevertheless, mung beans have been considered due to their comparatively low content of antinutrients, considerable amount of proteins (24 %), vitamins, minerals and bioactive compounds. Therefore, the use of mung beans resulted in enrichment of low-protein products and alleviation of protein malnutrition (*89*). Another study showed the potential of common beans (22.73 % protein content) as a protein supplement. Despite their nutritional benefits, common beans are beneficial in the treatment of coronary heart disease, diabetes, obesity and cancer (*81*, *90*-*92*). Although pigeon pea is underutilised due to its hard-to-cook nature, it is a good source of protein, fibre, vitamin B complex, minerals and has a low GI (*93*). Horsegram has been recognised as a potential ingredient in the formulation of commercial food products, especially bakery products or as a partial substitute in composite flours. The addition of horsegram improved the nutritional value of the products by increasing the protein, micronutrient and dietary fibre content. In addition, phenolic compounds, dietary fibre and complex carbohydrates help to reduce the GI and alleviate obesity, diabetes and heart diseases (*94*-*97*).

Although the addition of pulse flour improved the nutritional value and therapeutic properties of composite flour, dough viscosity and stability decreased because the dilution of gluten with the increasing amounts of pulse flour hindered the development of the gluten network. Specific volume and hardness of the formulated bread loaves were reduced by the increased proportion of pulse flour (*79*). Water absorption capacity of the composite flour was improved with the addition of red kidney bean flour, as it increased the fibre and protein content of the composite (*98*). Moreover, the addition of green gram flour also increased the water absorption capacity of the composites due to the high water-attracting polar amino residuals. The oil absorption capacity illustrates the ability of protein matrix to bind with fat *via* capillary action (*89*). Protein, which consists of hydrophilic and hydrophobic components, is the most important chemical compound that affects the oil absorption capacity (*98*). Better hydrophobicity as a result of increased non-polar amino acids exposed to fat with increasing protein content of the composite, increases the oil absorption capacity of the composite flours (*92*). Therefore, greater attention has recently been given to the use of pulses as a functional ingredient in various food products without affecting the desirable properties of the product and its eating quality (*94*).

### Roots and tuber crops

The most important root crops, namely cassava and sweet potato, and the most important tuber crops, particularly cocoyam, potato and yam, are consumed as staple or secondary staple crops. Root crops play a vital role in ensuring food security due to their inherent advantages and climatic resilience to extreme and unpredictable environmental fluctuations (*99*). Cassava has been recognised as a cheap and important source of carbohydrates that can be used as a promising substitute for wheat flour (*100*). Increased addition of cassava flour reduced the protein content of the composite flour due to its low protein content (*101*). Water absorption capacity, a favourable property in dough handling, is increased by the addition of high-quality cassava flour because the weak molecular arrangement and inadequate network architecture of cassava starch improve the penetration of water (*89*, *101*). Sweet potato can contribute to food security in developing countries as it has a short maturation period and can grow under unfavourable conditions. Moreover, it is an excellent source of β-carotene, minerals, vitamin C, B_1_, B_2_ and B_3_. Since sweet potato flour has a lower fat content (1.5 %) than whole wheat flour (3.90 %), it reduces the fat content of the final products, which has a positive effect on shelf life as it prevents rancidity. Products containing sweet potato flour provide the body with substantial amounts of fibre because sweet potato flour has a higher fibre content (3.28 %) than wheat flour (0.32 %) and helps to control the bowel integrity, reduce blood cholesterol and regulate blood sugar levels (*102*-*105*). Taro roots, which are easily digestible, are a better source of fibre (2.81 %), calcium and potassium (12 and 254 mg/100 g) than whole wheat flour (1.54 %, 0.217 and 1.762 mg/100 g, respectively). Functional properties of composite flour, including water and oil absorption capacity, increased with the addition of taro root. Taro can be used as an effective raw material in composite flours to improve the nutritional and functional properties of whole wheat flour (*106*). Cocoyam, an underutilised tuber, improved the nutritional value and affordability of wheat-based products (*107*). Cocoyam has a relatively higher mineral content (calcium, magnesium and phosphorus) and more digestible protein fraction than other important roots and tubers (*99*).

### Fruits and non-leafy vegetables

**Fig. 3:**
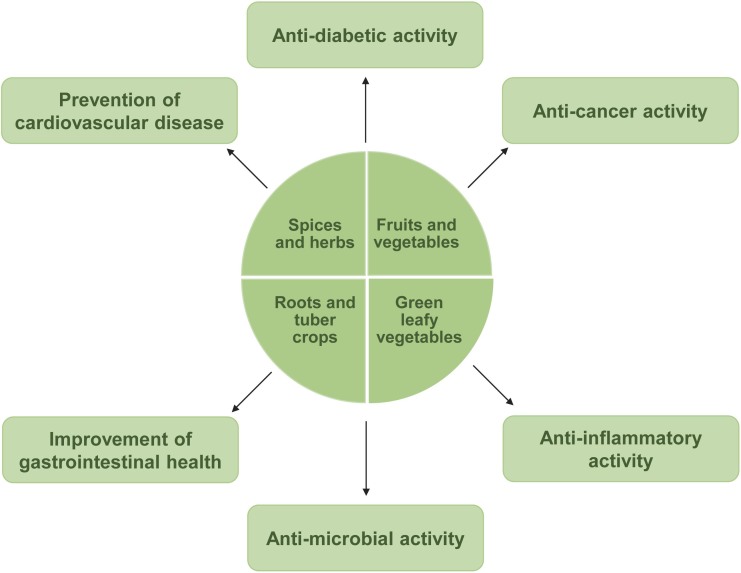
Health benefits of sustainable alternative flours (*109*-*112*)

Recently, greater attention has been paid to the use of natural raw materials for the development of functional foods that can positively affect human metabolism and promote healthy lifestyle through complete nutritional profile (*108*). Important health benefits of the main alternative plant sources other than cereals and legumes are shown in Fig. 3 (*109*-*112*). The bioactive compounds, the type of carbohydrates, the amino acid and fatty acid profiles of the natural food components can modify the food products to achieve an optimal nutritional balance. Vegetables have a beneficial effect on human health due to the presence of phyto-nutraceuticals, including minerals, vitamins and dietary fibre. Composite flour containing cauliflower can be recommended as a multifunctional flour mixture as cauliflower contains easily available bioactive compounds. Moreover, the addition of vegetables increases the ash content due to the high mineral content (*108*).

Avocado, known as a super-supplement due to its superior nutritional profile and numerous health benefits for humans, is used in the development of functional foods. The addition of avocado pulp improved the antioxidant activity, total phenolic, total flavonoid and carotenoid content, as well as high-quality fatty acid profile of whole wheat chapattis (*113*). Blending whole blackberry flour with whole grain wheat flour increased the total dietary fibre, total phenolic and anthocyanin content of the composite flour, improving its sensory properties, nutritional quality, antioxidant properties and health benefits (*114*).

### Mushrooms

Mushrooms are considered an unused food source of great culinary and therapeutic importance (*115*). The addition of mushroom to bread improved antioxidant activity and total phenolic content due to the higher content of glutathione and ergothioneine as antioxidants in mushrooms (*108*). Oyster mushrooms have been used as an ingredient in the development of functional cookies due to their therapeutic properties attributable to a wide range of bioactive compounds (*115*). They have shown antimicrobial and antioxidant properties as well as cytotoxic effects (*116*, *117*). Moreover, mixing oyster mushrooms increased the protein, fibre and ash content of whole wheat flatbread (*118*). In addition, powders of lion’s mane, turkey tail and reishi mushroom showed antimicrobial (*119*-*121*), antidiabetic (*122*-*124*), antiobesity (*125*-*127*), anticancer (*121*, *128*, *129*) and neuroprotective (*130*-*132*) properties, which could be due to numerous bioactive constituents with different configurations (*133*).

### Green leafy vegetables

Green leafy vegetables are cost-effective and nutrient-rich food that, in addition to their nutritional importance, contain a wide range of non-nutritive bioactive compounds that can have therapeutic properties. These vegetables are an excellent source of vitamins such as vitamin C, B_2_, E, folic acid and provitamin A compounds such as β-carotene and they contain significant amounts of minerals (*e.g.* iron, phosphorus and calcium). Therefore, the addition of green leafy vegetables plays an important role in nutrient deficiencies (*111*). The ash, protein, fibre, calcium, magnesium, zinc, iron, potassium and total phenolic content of moringa leaf flour are higher than of whole wheat flour. Moringa leaves also have antidiabetic (*134*), anticancer (*135*), antioxidant and antimicrobial properties (*136*). The addition of moringa leaves improved the antioxidant, nutritional and therapeutic properties of whole wheat flour (*137*). Spinach is considered as a naturally enriched green leafy vegetable with more ash (2.99 %), protein (19.18 %), fibre (8.19 %), sodium (98.20 mg/100 g), calcium (1303.9 mg/100 g), iron (40.36 mg/100 g) and zinc (13.38 mg/100 g) than whole wheat flour. Despite the improvement in nutritional and health benefits, the addition of spinach powder affected the rheology of the dough, while dough development and stability decreased with increasing amount of substitution (*138*). Green leafy vegetables have a known potential as functional ingredients that can be used to enrich or fortify food products (*111*).

### Herbs and spices

Spices themselves will not become a staple food, but they can improve the functional profile of food. The use of spices as flavour additives dates back thousands of years. Spices impart colour, flavour and aroma to foods while extending their shelf life. Spices and herbs have significant antioxidant properties due to phenolic compounds (*139*). The energy value, fat and protein content of composite flour increased with the addition of even a small amount of germinated fenugreek powder (5 %). Better nutritional and acceptable organoleptic properties of cookies baked at 175 °C were obtained by adding 10 % fenugreek flour (*140*). Dietary spices can also improve the digestive properties of food by stimulating the secretion of corresponding enzymes present in food. The addition of 1 and 2 % ajwain, cumin, cinnamon, black pepper, fennel and ginger increased the protein digestibility of whole wheat flour (*141*). Oregano and bay leaf improved the radical scavenging activity of whole wheat and meat-based bread. The high content of carnosic acid, rosmarinic acid and carnosol in oregano and the high content of methyl eugenol and eugenol in bay leaf could be responsible for their antioxidant potential. They also act as natural preservatives that reduce oxidative stress and microbial deterioration during storage (*142*). Saffron, considered a high-value spice worldwide, is not only used in cooking for its flavour and colour, but also has therapeutic properties including antidiabetic (*143*), cardioprotective (*144*), anticancer (*145*) and antidepressant effects (*146*, *147*). Therefore, spices can be used as functional ingredients to increase the nutritional value and medicinal properties of food (*139*).

### Nuts and seeds

Whole wheat muffins with added flaxseed are known as an appropriate functional food for people with cardiovascular disease and obesity (*148*-*150*). Chia seeds have high ratio of polyunsaturated fatty acids, α-linolenic acid (50–57 %) and a low ratio of linoleic acid/α-linoleic acid (LA/ALA). The lower the LA/ALA ratio, the greater the health benefits. Adding chia seeds to the diet helps prevent cardiovascular disease (*151*, *152*). A dietary fibre-enriched product can be made by mixing whole wheat flour with peony seed oil (15 g) and chia seeds (12 g) to improve antioxidant activity (*153*). The addition of prehydrated chia seeds or flour optimised the specific volume, texture and acceptability of the bread better than the untreated seeds or flour (*154*). The whole wheat biscuits with added walnuts have better shelf-life stability due to the higher antioxidant activity (*155*). The addition of cashew nuts to whole wheat composite bread resulted in an improved flavour and taste profile. Moreover, the addition of larger amounts cashew nut flour had a significant effect on the viscosity and consistency, resulting in an excellent mouth feel and overall sensory experience (*156*). Peanuts are known as an underutilised plant with high nutritional value. Peanut-enriched whole wheat flatbread has improved textural, antioxidant and sensory properties as well as higher macro- and micronutrient content (*157*).

### Fruit and vegetable side streams

Recently, the use of side streams from the food industry in the development of functional foods has attracted attention from an economic point of view as it reduces waste disposal costs, from a socioeconomic point of view as it improves nutritional value of products for consumers, and from an environmental point of view as it reduces waste disposal. Side streams include peels, seeds, juice and stems (*158*, *159*). The increased content of flavonoids and polyphenols as well as antioxidant activity of whole wheat flour with added date seed powder show that it is a functional food ingredient. Date seeds have been recognised as a promising source for averting liver damage and preventing hepatotoxicity in rats (*158*, *160*). The addition of jackfruit seed flour improved the nutritional value of the composite flour as it had higher ash (3.4 %), protein (13.3 %), fibre (5.1 %), calcium, magnesium, potassium and phosphorus content (68.4, 161.6, 1454.4 and 301.7 mg/100 g, respectively) than whole wheat flour. In addition, bioactive and phytochemical ingredients have antidiabetic (*161*), antioxidant (*162*) and anti-inflammatory (*163*) properties, which provide various health benefits to humans (*164*). Orange peels contain multiple bioactive compounds, including flavonoids and cinnamic acids, and have various health-promoting properties, such as anticarcinogenic (*165*), anti-obesity (*166*) and anti-diabetic (*167*) properties. Despite the fact that apple and cherry tomato peels have significant health benefits, the addition of these peel flours to whole wheat flatbread led to the formation of acrylamide (*133*). Another study reported increased nutritional value and mineral content (*i.e*. iron, potassium, calcium, sodium and magnesium) of whole wheat cookies with added avocado/banana peel (*168*). Apple pomace, Indian gooseberry pomace powder and bottle gourd peels were identified as potential alternatives in whole wheat composite flour because of their higher content of ash, fibre, pectin, vitamin C, total phenols and better health benefits (*159*).

### Microalgae

Recently, there has been an increased focus on the supplementation of microalgae, as they contain promising bioactive compounds that can act as functional components (*169*). Microalgal biomass is widely used as dietary supplements, herbal products and nutrient isolates. In addition to the direct consumption of microalgal biomass, they can be used in novel food formulations due to their balanced chemical composition, especially their versatile fatty acid profiles, antioxidants, vitamins, minerals and high-quality proteins, as well as their specific interesting properties (*169*, *170*). The integration of microalgae into the diet confers therapeutic properties such as cardioprotective, anticancer, antioxidant, immunomodulatory and chemoprotective properties (*171*).

Spirulina, a blue-green filamentous microalga, is an excellent source of protein (60–70 %) with high biological importance due to its vitamins (vitamin B_12_ and provitamin A), minerals (iron, calcium and magnesium) and bioactive compounds (total phenols, chlorophylls, carotenoids and flavonoids) (*169*, *171*). They have antioxidant (*172*), antidiabetic (*172*), immunomodulatory (*173*), antihypercholesterolaemic (*174*), anticancer (*175*) and anti-inflammatory activity (*175*). Spirulina has been recognised as a potent candidate for functional foods due to the above benefits. It has increased the protein digestibility of whole wheat flour bread (*170*). Moreover, spirulina has contributed to the improvement of the amino acid profile, nutritional value and bioactive parameters of whole wheat pasta (*171*). *Nannochloropsis* microalgae are known for their higher content of eicosapentaenoic acid (EPA), which is beneficial in biological membrane functions. The addition of *Nannochloropsis* to the pasta significantly increased the protein, ash, lipid and EPA acid content (*176*). The combination of *Haematococcus pluvialis*, astaxanthin and dietary fibre-rich marine-derived microalgae significantly reduced the rate of glucose release during *in vitro* digestion of whole wheat cookies. The addition of microalgae thus reduced the glycaemic response and improved the bioactive compounds of whole wheat cookies (*177*).

## APPLICATION OF COMPOSITE FLOURS IN FOOD INDUSTRY

There is an increasing interest in the successful use of composite flours in the development of a wide range of food products, especially bakery products, with the intention of improving the functional and technological properties of the final product. Different amounts of different types of flour are mixed with wheat flour to develop bakery products. The amount to be replaced is determined by the quality and quantity of wheat proteins, as it determines the desired degree of dough viscosity, plasticity and elasticity, to obtain the quality of the final product (*100*, *178*).

The use of different food components in product formulations is determined by the functionality of each ingredient, *i.e.* the properties other than nutritional value that regulate the behaviour of foods during different treatments (*89*). For instance, too high or too low water absorption capacity of flours has a detrimental effect on product quality. A higher water absorption capacity is preferred for the development of texture of bakery products such as bread and biscuits (*92*). Water absorption has a positive effect on loaf volume, fracture stress of bread crumb, proofing and bread yield. Excessive water absorption leads to a large bread volume, an open crumb with oversized cells and an increased susceptibility to mould, while insufficient water absorption leads to a small bread volume with a firm and dense crumb structure (*98*). A higher oil absorption capacity of composites leads to a better flavour, mouthfeel, texture of the food and a longer shelf life, especially of bakery products (*89*, *92*).

The bakery products made from composite flour were of good quality with improved nutritional value and appearance. Despite the fact that composite flour products have some similar properties to wheat flour products, there were differences between the two derivatives in terms of textural, functional and sensory properties. Previous studies emphasised the need for additional efforts to improve the bread quality with higher additions of gluten-free flours. The gluten content and the viscosity of the starch determine the influence of alternative flours on the wheat addition and thus on the baking quality. Physical properties of the formulated bread, particularly loaf volume, mass and specific volume, are bread quality attributes that evaluate the effect of the alternative flours (*89*). The addition of alternative flours reduced the amount of gluten in the composite flours, resulting in reduced carbon dioxide retention and ultimately a reduction in the height, loaf volume and specific volume of the composite breads. Moreover, the loaf volume decreased with an increasing amount of legume flours, as the comparatively larger particles of legume flour can penetrate gas cells during dough expansion. The size of the baked dough, the moisture and the amount of CO_2_ gas diffused from the loaf during baking determine the bread mass. Composite breads have a higher loaf mass due to the lower CO_2_ retention. The specific volume has been recognised as a credible measure of loaf size. The minimum hydration capacity relevant for composite flours could be the reason for the lower specific volume (*93*). Greater quantities of open crumb pores and larger crumb cell walls were observed with the increasing amount of pulse flour. Bread hardness increased with the higher proportion of pulse flour due to the increased starch retrogradation (*79*).

Physical parameters related to the cookies are mass, thickness, diameter and spread ratio. A higher amount of alternative flours with the ability to increase the amount of water-absorbing fibre reduced the spread factor of the cookies, resulting in cookies with a smaller diameter and higher thickness. The robust protein interaction between the individual flours of the composite mixture determined the hardness of the cookies (*46*). In addition, the interaction between gluten and fibre affected the hardness of the cookies, as the dietary fibre content increased the water absorption capacity and influenced the gluten development time. Higher amounts of date seed powder reduced the hardness of the cookies due to the high dietary fibre content (*158*). The spread ratio of the cookies and their fat content are known to be directly corelated. The researchers observed a reduction in the spread ratio of the cookies by adding crude lycopene, a fat-soluble component, which reduced the amount of free fat available for wheat flour. Furthermore, the addition of tomato powder was observed to increase the hardness of the cookies due to the increase of fibre content (*179*). Previous studies reported increased mass in composite cookies due to the higher moisture content of fenugreek and higher bulk density of the flour mixtures. Moreover, the higher protein content of oats and fenugreek reduced the spread ratio of the cookies (*140*).

The cooking properties determined the quality of the developed pasta. Fractionated whole wheat and bambara ground composite pasta had a shorter optimum cooking time (OCT) than the control pasta made from unfractionated flour. Fractionated flours had shorter OCT because they contained larger particle sizes. Larger particles, particularly germ and fibre, facilitate water absorption and reduce the preparation time (*180*). Higher insoluble dietary fibre (IDF) amounts prevent complete gelatinisation of starch, as IDF competes with starch for water and ultimately shortens cooking time and thus saves energy. Cooking loss is another critical quality parameter for pasta. The higher the cooking loss, the lower the quality of the pasta. Smaller quantities of alternative flours cannot considerably weaken the gluten-starch matrix and therefore have no significant effect on cooking loss. The addition of high-fibre alternative flours reduced the hardness of the pasta by weakening the gluten matrix of the pasta structure (*181*). Some research on the successful use of composite flours in the food industry is shown in Table 1 (*19*, *45*-*47*, *55*-*57*, *66*, *79*, *82*, *89*, *92*-*94*, *98*, *100*-*102*, *107*, *108*, *115*, *137*, *138*, *140*, *142*, *147*, *148*, *151*, *158*, *168*, *179*, *180*, *182*-*198*).

**Table 1:** Application of composite flours in food industry

Product	Composite flour	Acceptable ratio	Specific nutritive properties	Specific functional properties	Ref.
Bread	Whole wheat/yellow pea	95:5	Increased ash and protein content of the flour mixture reported due to the incorporation of yellow pea	Improved rheological properties (*i.e*. handling, mixing and pasting properties) compared to whole wheat flour dough The specific volume and bread quality are comparable with whole wheat bread	(*79*)
	Whole wheat/acha/pigeon pea/date palm fruit sugar	60:20:20:100	Increased protein, crude fibre and mineral content (*i.e*. Na, K, Ca, Mn, Zn) Considerable phytate content and lower amount of oxalate Low glycaemic index	Reduced specific volume, volume and height, and increased mass (*i.e.* denser bread)	(*93*)
	Whole wheat/watermelon seed	97.5:2.5	Increasedprotein and fibre content and lower carbohydrate and ash content Increased minerals (*i.e*. iron, phosphorous and magnesium) Lower tannin and oxalate content	Improved loaf mass, volume and decreased specific volume	(*182*)
	Whole wheat/sweet lupine	75:25	Increased protein and mineral content (specifically calcium and zinc)	-	(*183*)
	Whole wheat/chickpea	60:40	Reduced glycaemic response	Reduced specific volume	(*82*)
	Whole wheat/cassava/green gram	90:5:5	Increased fat, protein and fibre content.	Increased loaf mass, reduced volume and specific volume, sensory properties comparable to 100 % whole wheat bread	(*89*)
		80:10:10	Elevated ash, fat, protein and fibre content.	Increased loaf mass, volume and specific volume. Comparable with 100 % wheat bread	
	Whole wheat/chicken meat powder/amaranth/oregano/bay	59.50:30:10: 0.5	Enhanced antioxidant activity and storage stability	Enhanced overall acceptability and structural integrity during storage compared to whole wheat bread	(*142*)
	Whole wheat/chia seed/quinoa/amaranth	67:10:4:19	Increased soluble and insoluble dietary fibre, total dietary fibre, ash and protein content Reduced caloric value and GI	Specific volume, shape ratio and crumb structure 100 % comparable to whole wheat bread	(*151*)
	Whole wheat/cocoyam/bambara ground	70:18:12	Elevated fibre, protein and ash content	Comparable with 100 % whole wheat bread with respect to sensory properties	(*184*)
	Whole wheat/mutamba fruit flour	95:5	Improved bioactive content and reduced caloric value in the bread	Loaf volume reduced with the increasing amounts of substitution	(*185*)
	Whole wheat/brown seaweed	96:4	Increased ash and total dietary fibre content	Soft and chewy bread with no significant aftertaste	(*186*)
	Whole wheat/red seaweed	98:2	Increased ash, protein and total dietary fibre content		
	Whole wheat/red kidney bean/defatted coconut flour	90:5:5	Increased protein, ash, fat, fibre, phytate, oxalate and tannins content	Hard crumb texture	(*98*)
	Whole wheat/sorghum/millet (prefermented in the presence of exopolysaccharides)	50:50	Enhanced nutritional value. Reduced GI	Hard texture and brown coloured crumb structure	(*45*)
	Whole wheat/cassava	90:10 80:20	Increased ash content	Promoted gumminess, cohesiveness and resilience of bread	(*100*)
	Whole wheat/cocoyam	Acceptable: 85:15	Increased content of fibre, ash and fat	Reduced textural attribute of bread due to the reduction of dough elasticity with the increasing amounts of substitution	(*107*)
		Most acceptable: 95:5			
	Whole wheat/vegetable paste (mushroom/cauliflower/pea)	85:15	Increased ash, protein and total phenolic content Enhanced antioxidant activity	Reduced loaf volume and increased hardness	(*108*)
	Whole wheat/moringa leaf powder	95:5	Increased ash, protein, fibre content and calories. Calcium, potassium, magnesium, zinc contents and anti-oxidant capacity improved	Increased gumminess and reduced hardness and springiness of bread	(*137*)
Flat bread	Whole wheat/stabilised rice bran/undamaged- stabilised-debittered wheat germ	75:10:15	Increased protein, ash and total dietary fibre content and lowered starch digestibility	Improved water absorption capacity, dough mixing and handling properties	(*187*)
	Whole wheat/extruded finger millet	80:20	Increased dietary fibre, protein, iron and calcium	Improved extensibility and reduced resistance to extension (*i.e*. soft and less firm flatbread)	(*188*)
	Whole wheat/barley	80:20	Increased content of ash, protein, β-glucan and energy and reduced carbohydrate content	Improved water absorption capacity, dough development time and reduced dough stability	(*57*)
	Whole wheat/fenugreek gum	99.25:0.75	Increased moisture content	Sensory qualities similar to 100 % whole wheat flatbread. Excellent pliability and softness observed up to 2 days of storage	(*189*)
	Whole wheat/barley	75:25	Increased crude fibre, ash content and reduced fat and protein content Low glycaemic index	No significant difference in sensory properties with up to 25 % barley flour addition Reduced extensibility and force to tear	(*55*)
	Whole wheat/broken rice flour	80:20	Higher digestible starch and lower resistant starch Increased GI	Dough development time and stability increased Reduced shrinkage and bake loss	(*19*)
	Whole wheat/sorghum/pearl millet	40:30:30	Reduced GI	Improved hardness and stiffness	(*47*)
	Whole wheat/spinach	92.5:7.5	Increased protein, ash, fiber, potassium, calcium and iron content	Reduced water absorption, dough development time and stability Enhanced hardness, springiness and chewiness Reduced puffed height	(*138*)
Cookies	Whole wheat/common bean/pumpkin	75:15:10	Increased protein, fat, ash and crude fibre contents	Reduced lightness and specific volume Increased spread ratio	(*92*)
	Whole wheat/pearl millet/sorghum	30:40:30	Reduced GI	-	(*47*)
	Whole wheat/horse gram flour	75:25	Enhanced nutritional value	Increased spread ratio and reduced mass, diameter and thickness No adverse effect on the overall acceptability	(*94*)
	Whole wheat/soy okara/tigernut	60:20.98:19.02	Increased fibre ash, magnesium, iron and sodium content	No significant difference in mass, diameter, height, appearance, mouthfeel, crunchiness and crispiness Significant difference in spread ratio and thickness	(*190*)
	Whole wheat/sorrel seed protein isolate/cassava flour	85:5:15 70:10:20 55:15:30	Significantly increased crude protein, fat, fibre, ash, mineral content Reduced carbohydrate content Increased mineral content with increased substitution	No significant difference in the sensory attributes of cookies, except crispiness at all level of inclusion Improved pasting and functional properties	(*101*)
	Whole wheat/unripe plantain/germinated pumpkin seed	90:5:5	Significantly increased crude protein, fat, fibre and ash content Reduced carbohydrate. Increased tannins, oxalates and phytates	No significant difference in spread ratio compared to whole wheat cookies	(*191*)
	Malted whole wheat/malted coarse grain (barley, sorghum, pearl millet) blend/defatted soy flour	50:40:10	-	Enhanced functional properties	(*192*)
	Whole wheat/pearl millet flour	40:60	Increased fat, fibre, iron, calcium and phosphorus content	Significantly reduced mass, diameter and spread factor. Improved hardness, chewiness, gumminess, breaking and cutting strength	(*46*)
	Whole wheat/cladode flour	75:25	Increased total phenolic content Enhanced radical scavenging activity	No significant difference in mass, diameter and hardness Significant difference in thickness and spread ratio	(*193*)
	Whole wheat/date seed powder	92.5:7.5	Enhanced total phenolic content, flavonoids and antioxidant capacity	Enhanced crispiness	(*158*)
	Whole wheat/tomato powder	98:2	Enhanced antioxidant activity, reducing power, total carotenoid and total phenolic content increased ash and fat content	Better rising ability of cookies due to reduced spread ratio	(*179*)
	Whole wheat/crude lycopene	99.9:0.1			
	Whole wheat/germinated pumpkin seed flour	70:30	Increased ash, protein, fat, mineral (*i.e*. calcium, magnesium iron and zinc) and total dietary fibre (*i.e.* soluble and insoluble) content	Reduced diameter and thickness Increased spread ratio	(*194*)
	Whole wheat/germinated fenugreek seed flour/oats	70:10:20	Increased protein, crude fibre, fat, ash, mineral (*i.e*. calcium, magnesium, zinc and iron) and energy content Reduced carbohydrate content Moderately increased anti-nutrient content including phytic acid and condensed tannins	Reduced spread ratio and crispiness Improved mass	(*140*)
	Whole wheat/saffron	99:1	Enhanced resistant starch content, total phenolic and DPPH radical scavenging activity	Improved lightness, hardness and spread factor	(*147*)
	Whole wheat/quinoa seed flour	60:40	Nutritionally acceptable ratio due to presence of the highest protein, fibre and ash contents Reduced carbohydrate and fat content	Improved spread ratio Reduced width and diameter	(*66*)
		90:10	Organoleptically highest acceptable ratio Increased protein, fibre and ash content compared to 100 % whole wheat biscuits	Reduced spread ratio and diameter Improved width	
	Whole wheat/avocado peel	95:5	Increased protein, fibre, ash (*i.e.* potassium, calcium, sodium, magnesium and iron)	Enhanced crispiness	(*168*)
	Whole wheat/amaranth/nopal/oyster mushroom	50: 30:15:5	Increased ash, fibre and protein Enhanced total phenolic, total flavonoid content and antioxidant activity	No adverse effect on sensorial attributes	(*115*)
Noodles	Whole wheat/sorghum/chia seed	-	-	Improved cooking qualities, water absorption capacity and mass of the noodles Reduced cooking losses	(*195*)
	Whole wheat/unripe banana flour	55:45	Increased fibre, and resistant starch content	Reduced cooking time Improved water absorption and rehydration ratio	(*196*)
	Whole wheat/foxtail millet/mushroom/rice bran	40:50:5:5	Increased ash, protein, fat and fibre content Reduced carbohydrate content Improved amino acid profile Excellent source of calcium, iron and phosphorous	Improved organoleptic properties including flavour and taste	(*197*)
	Whole wheat/potato peel flour	60:40	Possess considerable amount of energy Improved nutritive value	Optimum ratio for 3D printed noodles with the adequate strength to withstand post-processing steps, better pasting properties, flowability and printability Acceptable sensory properties. Cooking quality and textural properties comparable to the commercial product	(*198*)
	Whole wheat/oat flour	70:30	-	Improved hardness and chewiness Increase in hardness of noodles with the increasing dough sheet thickness. Reduced cooking time Decrease in cooking time with the reduced thickness of the noodle	(*56*)
Pasta	Whole wheat/bambara groundnut	80:20	Increased fat, protein, ash contents and reduced carbohydrate and fibre content with the reduction of particle size Reduced total phenolic content and increased antioxidant activitiy in fractionated flour pasta with the reduction of particle size	Increased optimum cooking time with reduced particle size Comparatively higher scorings for sensory attributes in terms of colour, taste, mouthfeel and overall acceptability of fractionated flour pasta with finer particles	(*180*)
Cakes/ muffins	Whole wheat/sweet potato/pigeon pea	70:10:20	Increased fibre, protein and ash (*i.e*. Ca, P and K) content Reduced fat content Increased tannin and trypsin inhibitor Reduced phytate content	Organoleptically comparable with the whole wheat cakes	(*102*)
	Whole wheat/ungerminated flaxseed	85:15	Increased protein, ash and fibre content	Improved softness Reduced mass and volume	(*148*)
	Whole wheat/germinated flaxseed	90:10			

## BIOAVAILABILITY AND BIOACCESSIBILITY STUDIES OF WHOLE WHEAT-BASED FUNCTIONAL FOODS

There is a growing trend of consumer preference for native food components and natural products over synthetic compounds to achieve the desired health benefits through a regular diet (*199*). Despite the presence of numerous bioactive molecules in foods, the consumption of these foods is not necessarily associated with favourable health effects (*200*). Furthermore, not all bioactive substances are utilized efficiently by the organisms (*199*). Therefore, studies to evaluate the bioactivity of functional foods and their nutritional efficacy are essential (*199*, *200*). Factors affecting the efficacy of bioactive compounds in foods include their steadiness in the food matrix, bioavailability, metabolomics and nutrigenomics. The crucial characteristic of any food formulation is the bioavailability of its nutrients (*199*). From nutritional point of view, bioavailability is defined as the proportion of a nutrient that is available either for physiological activities or storage. First, a food component must be released from the food matrix and digested to become available. For this reason, bioaccessibility is the preliminary step of bioavailability. It is defined as the amount of nutrients released from the food matrix into the gastrointestinal tract and enter the bloodstream in a suitable form for absorption (*199*, *200*). Bioavailability and bioaccessibility studies of whole wheat-based functional foods are listed in Table 2 (*47*, *82*, *93*, *141*, *147*, *151*, *201*-*209*).

**Table 2:** Bioavailability and bioaccessibility studies on whole wheat-based functional foods

Product	Composite flour	Study type	Outcome	Ref.
Bread	Whole wheat/green coffee	*In vitro*	Increased bioaccessibility of phenolic compounds compared to control whole wheat bread	(*201*)
	Whole wheat/quinoa	*In vivo*	Reduced triglyceride, total cholesterol, low-density lipoprotein (LDL) and very-low-density lipoprotein (VLDL) levels Reduced GI (42.0) compared to control (69.20)	(*202*)
	Whole wheat/amaranth/acha	*In vitro*	Enhanced metal chelating and radical scavenging ability Higher α-amylase and α-glucosidase inhibitory activity Reduced GI	(*203*)
	Whole wheat/acha/ pigeon pea/date palm fruit sugar	*In vivo*	Reduced GI Reduced postprandial blood glucose response	(*93*)
	Whole wheat/chickpea	*In vivo*	Reduced glycaemic response	(*82*)
	Whole wheat/quinoa/ chia/amaranth	*In vitro*	Reduced GI (85.0) compared to control (95.0) Reduced protein digestibility Increased nutritional index	(*151*)
Flatbread	Whole wheat/moringa/ C_4_H_2_FeO_4_	*In vitro*	Higher mass ratio of bioaccessible iron and calcium (2.75 and 50.09 mg/100 g) than control (0.16 and 53.0 mg/100 g) Higher mass ratio of bioaccessible carotene and β-carotene (0.203 mg/100 g and 183.04 µg/100 g) than control [not detected (ND)]	(*204*)
	Whole wheat/ moringa/FeSO_4_		Higher mass ratio of bioaccessible iron and calcium (2.54 and 47.19 mg/100 g) than control (0.16 and 53.0 mg/100 g) Higher mass ratio of bioaccessible carotene and β-carotene (0.226 mg/100 g and 190.86 µg/100 g) than control (ND)	
	Whole wheat/ amaranth leaves/ C_4_H_2_FeO_4_		Higher mass ratio of bioaccessible iron and calcium (3.77 and 71.39 mg/100 g) than control (0.16 and 53.0 mg/100 g) Higher mass ratio of bioaccessible carotene and β-carotene (0.157 mg/100 g and 158.91 µg/100 g) than control (ND)	
	Whole wheat/ amaranth leaves/ FeSO_4_		Higher mass ratio of bioaccessible iron and calcium (3.87 and 75.0 mg/100 g) than control (0.16 and 53.0 mg/100 g) Higher mass ratio of bioaccessible carotene and β-carotene (0.150 mg/100 g and 156.28 µg/100 g) than control (ND)	
	Whole wheat/ chickpea/FeSO_4_		Higher mass ratio of bioaccessible iron (3.39 mg/100 g) than control (0.16 mg/100 g)	
	Whole wheat/ chickpea/C_4_H_2_FeO_4_		Higher mass ratio of bioaccessible iron (2.16 mg/100 g) than control (0.16 mg/100 g)	
	Whole wheat/green gram/FeSO_4_		Higher mass ratio of bioaccessible iron and calcium (3.38 and 81.47 mg/100 g) than control (0.16 and 53.0 mg/100 g)	
	Whole wheat/green gram/C_4_H_2_FeO_4_		Higher mass ratio of bioaccessible iron and calcium (2.43 and 81.14 mg/100 g) than control (0.16 and 53.0 mg/100 g)	
	Whole wheat/buckwheat	*In vitro*	Increased *in vitro* protein digestibility from 78 to 88 % with the addition of buckwheat flour	(*205*)
	Whole wheat/spices (ajwain/ cumin/cardamom/ fennel/cinnamon/ginger/black pepper)	*In vitro*	Enhanced protein digestibility Increased bioaccessibility of iron and zinc	(*141*)
	Whole wheat/pigeon pea	*In vitro*	Reduced slowly digestible starch and increased resistant starch content Reduced starch digestibility Reduced predicted glycaemic index High protein digestibility	(*206*)
	Whole wheat/fenugreek	*In vitro*	Reduced starch digestibility (27.12 %) compared to control (37.87 %) Reduced protein digestibility (83.56 %) compared to control (68.18 %)	(*207*)
	Whole wheat/sorghum/pearl millet	*In vivo*	Reduced GI (25.40) compared to control (40.40) Showed hypoglycaemic effects	(*47*)
Biscuits/cookies	Whole wheat/sorghum/pearl millet	*In vivo*	Reduced GI (27.50) compared to control (44.28) Hypoglycaemic effects	(*47*)
	Whole wheat/wheat germ/coffee silver skin	*In vitro*	Increased phenolic bioaccessibility compared to control whole wheat cookies Increased antioxidant bioaccessibility of cookies compared to control	(*208*)
	Whole wheat/saffron	*In vitro*	Increased resistant starch content Reduced starch digestibility	(*147*)
	Whole wheat/buckthorn			
Bagels	Whole wheat/banana peel/lavender	*In vivo*	Reduced anxiety score among participants Anxiolytic potential	(*209*)

## CONCLUSIONS

Composite flour technology plays a vital role in improving the economic status of a country by saving the foreign exchange provided for wheat import and promoting the local agriculture by using indigenous crops, which increases rural employment and income. Partial substitution of wheat flour is the most feasible approach of the composite flour technology because it can complement the nutritional profile of wheat. Recently, consumer interest in non-wheat flour has increased due to its ability to ameliorate non-communicable diseases, which is due to the presence of macronutrients, micronutrients and phytochemicals in significant amounts. The results of research studies have shown that the deficiency in micro- and certain macronutrients can be eliminated by the objectively formulated composite flour-based products. The inclusion of leafy, non-leafy vegetables, roots and tubers along with fruits in the composite flour formulations is expected to provide numerous nutritional benefits. The phytochemicals available in these sources not only have a positive effect on health, but also improve the physicochemical properties of the final product. When the components with different properties are combined together in the formulation of composite flour, the rheological properties of the dough are improved in different ways. Since most of the aforementioned substances are underutilised or unidentified as highly nutritious sources, the use of these materials in composite flour formulations leads to a reduction in the cost of flatbread and other bakery products, thus ensuring the country’s national food security. The use of composite flours in the food industry has paved the way for the formulation of products with significant nutritional, organoleptic and therapeutic properties. The appropriate quality and quantity of flour combinations can be determined according to product technology, consumer requirements and acceptance. However, there is still much room for scientific research to evaluate the process modifications, novel methods and additional sources that can optimise the quality of the composite flour products. This will lead to the popularisation of the composite flour products and capturing a significant market in the near future due to their economic value, health benefits and nutritional properties.
